# The long-term residual effects of low-magnitude mechanical stimulation therapy on skeletal health

**DOI:** 10.1186/s13036-020-0232-x

**Published:** 2020-03-14

**Authors:** Kyle A. Bodnyk, Kyle S. Kuchynsky, Megan Balgemann, Brooke Stephens, Richard T. Hart

**Affiliations:** Department of Biomedical Engineering, The Ohio State University, 1080 Carmack Rd, Columbus, OH 43210 USA

**Keywords:** Skeletal health, Dynamic histomorphometry, Micro-CT, Bone mineral density, Murine model, Osteoporosis, Preventative medicine

## Abstract

**Background:**

Low-magnitude mechanical stimulation (LMMS) may improve skeletal health. The objective of this research was to investigate the long-term residual effects of LMMS on bone health. 10-week old female mice were given LMMS for 8 weeks; SHAM did not receive LMMS. Some groups remained on study for an additional 8 or 16 weeks post treatment (*N* = 17).

**Results:**

Epiphyseal trabecular mineralizing surface to bone surface ratio (MS/BS) and bone formation rate (BFR/BS) were significantly greater in the LMMS group compared to the SHAM group at 8 weeks by 92 and 128% respectively. Mineral apposition rate (MAR) was significantly greater in the LMMS group 16 weeks post treatment by 14%.

Metaphyseal trabecular bone mineral density (BMD) increased by 18%, bone volume tissue volume ratio (BV/TV) increased by 37%, and trabecular thickness (Tb.Th.) increased by 10% with LMMS at 8 weeks post treatment. Significant effects 16 weeks post treatment were maintained for BV/TV and Tb.Th. The middle-cortical region bone volume (BV) increased by 4% and cortical thickness increased by 3% with 8-week LMMS.

**Conclusions:**

LMMS improves bone morphological parameters immediately after and in some cases long-term post LMMS. Results from this work will be helpful in developing treatment strategies to increase bone health in younger individuals.

## Background

Osteoporosis is a common degenerative bone disease and is characterized by a net loss of bone, e.g., when bone is resorbed more quickly than it is formed. It primarily affects the elderly and is most common in women. 50% of women living in the United States over the age of 65 will develop osteoporosis. Treatment in the United States alone cost 18 billion in 2002 and that cost is projected to double or triple in the next few decades [1, 2]. The risk for a 50 year old women to develop a spine, wrist, or hip fracture in her lifetime is 39.7% [3]. Adults and children with impairments of musculoskeletal function such as cerebral palsy, muscular dystrophy, and spinal cord injury are also susceptible to osteoporosis [4–9].

Current drugs used to treat osteoporosis may work to either increase bone formation or to decrease bone resorption. As an alternative to drug therapy, whole body vibration (WBV) may improve skeletal health without potential side effects from drugs such as osteonecrosis of the jaw [10]. This therapy is administered through standing on an oscillatory platform that delivers low intensity vibration (LMMS). These vibrations are composed of low magnitude (0.3 g) accelerations at high frequency (20–50 Hz). LMMS may induce mesenchymal stem cells (MSC) to differentiate into osteoblasts rather than adipocytes [11]. These signals may also drive hematopoietic stem cells (HSC) to differentiate into immune cells rather than osteoclasts [12]. This combination of increasing osteoblasts and decreasing osteoclasts could contribute to bone formation and improved skeletal health [12].

In one clinical study, young women (15–20 years) with low BMD were given WBV (0.3 g, 30 Hz, 10 min/day) for 1 year. Compared to the control group that did not experience WBV, there was a 2.0% increase in vertebral trabecular bone and a 2.3% increase in femoral midshaft cortical bone [13]. These results suggest WBV promotes a healthy musculoskeletal system, which could improve long-term resistance to osteoporosis. Another one-year clinical study of postmenopausal women found femoral neck BMD in the WBV group to be 2.17% greater compared to placebo group [14]. In that study, WBV only prevented BMD loss; the anabolic benefits observed during the above study of young women given WBV, were not observed with postmenopausal women [14]. LMMS has also been studied as a potential therapy to reduce bone loss for people with spinal cord injuries, end-stage renal disease, and pediatric Crohn’s disease [15, 16].

In addition to osteoporosis during aging, people with disabilities can have reduced bone health. Specifically children with disabilities such as cerebral palsy, muscular dystrophy, spinal cord injury, pediatric Chron’s disease, and other diseases that affect ambulation can have reduced bone health and strength [4–9, 17]. Bone strength is reduced in children with these disabilities compared to non-disabled children, which results in increased fractures and occurrence of spontaneous fractures [4, 6, 8, 18]. One study of children with cerebral palsy ages 2–19 found a fracture incidence of 4% per year [19]. Yearly fracture rate of able bodied children under the age of 16 has been found to be as 1.6–1.8% [20, 21].

In order to establish WBV as a therapy for osteoporosis and other diseases, long-term implications must be studied. LMMS clinical studies monitoring BMD greater than 1 year have not been published, so the long-term efficacy of WBV is unknown.

Animal models resolve some issues with clinical trials and allow for more precise ex vivo imaging modalities, compliance, and variable control. In one study, a mouse model was used to observe the influence of LMMS on trabecular and cortical bone formation and resorption. Reproductively mature 8-week old mice were given LMMS (45 Hz, 0.3 g, 15 min/day) therapy for 3 weeks. Bone formation was 30% greater in the endocortical metaphysis for the LMMS group compared to age-matched controls. In the LMMS group, osteoclastic activity was reduced by 30% in the epiphysis and metaphysis compared to age-matched controls [22]. Significant morphological differences were not found during this 3 week study, likely attributed to the short study duration [22].

Referencing the above 3-week study, mice were given LMMS treatment for 6 weeks. Results indicated mineralizing surface to bone surface ratio (MS/BS) in the trabecular metaphysis were 75% greater in the WBV group compared to age-matched control group [23]. LMMS increased cortical bone area by 11%, bone marrow area by 12%, polar moment of inertia by 25%, and maximum moment of inertial by 29% [23]. These results indicate increased trabecular and cortical bone apposition in a reproductively mature mouse model compared to control.

Due to the short duration of the previous studies, the long-term benefits from LMMS therapy are unknown. The objective of this research is to investigate longer term effects of whole body vibration therapy on bone health and strength, and its potential efficacy as a preventative treatment for osteoporosis due to aging or ambulation disabilities. Using a mouse model, LMMS was applied for study of time-dependent variations of cortical and trabecular bone morphological parameters, and osteoblast activity.

We hypothesized that LMMS treatment would improve trabecular and cortical bone morphological parameters including BMD, compared to age matched controls during and after LMMS therapy. To compare, we made μCT scans of the femurs at different time points after LMMS therapy. Reconstructed images were analyzed yielding morphological metrics and BMD of specific cortical and trabecular bone regions of interest throughout the femur.

A second hypothesis was to see if LMMS treatment would increase bone apposition rate compared to age matched controls during and after LMMS therapy. To compare, fluorochrome histomorphometry of the femurs was be performed at various time points after LMMS therapy. Femur sections were imaged through florescent light microscopy to highlight areas of florescent label concentration and used to calculate bone apposition rate as well as new bone formation percent area. A three dimensional image was compiled for each bone, that allowed for new bone formation percent volume and highlighted sections of bone undergoing remodeling.

## Materials and methods

**Fig. 1:**
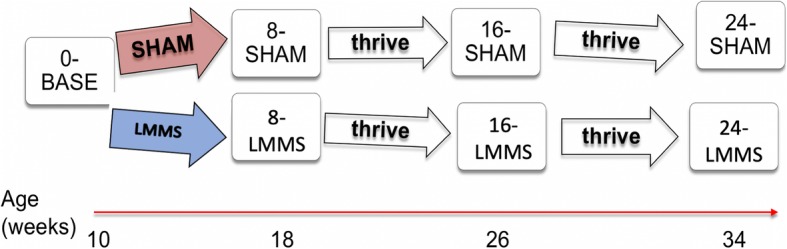
Mouse study design. For LMMS groups, treatment time was always 8 weeks

All animal care and procedures were approved by OSU IACUC #2016A00000099. 119, 10-week old female BALB/cByJ [23] mice were selected for the study. 10-week mice were selected for the study because mice are reproductively mature at that age, but not skeletally mature [22, 23]. By the end of the study, animals were skeletally mature and approaching or at middle age [24]. Animals were randomized into 4 or 5 animals per cage. Experimental mice were given LMMS for 8 weeks while control mice received SHAM treatment. Some groups stayed on study for an additional 8 or 16 more weeks but did not receive any treatment. Mice were euthanized at 8-week intervals throughout the study, and both left and right femurs were used for analysis. Groups consisted of baseline controls (0-BASE), 8-week LMMS (8-LMMS), 8-weeks post-LMMS (16-LMMS), 16-weeks post-LMMS (24-week), 8-week SHAM (8-SHAM), 8-weeks post-SHAM (16-SHAM), and 16 weeks post-SHAM (24-SHAM), for a total of 7 groups (*N* = 17). A visual representation of the study design is shown in Fig. 1. Sample size was determined through prospective power analysis α = 0.05 and power = 0.9, with the sample size validated by a statistician.

### LMMS

Experimental mice received LMMS through a vibration platform for 15 min a day, 5 days a week, for 8 weeks [22, 23, 25]. This platform produced 0.3 g acceleration signal at 90 Hz [22, 23]. SHAM mice were placed on the LMMS platform powered off. At the completion of the treatment period, femora were dissected, soft tissue was removed, and the bones were wrapped in saline soaked gauze, and stored at − 20 °C.

### MicroCT

Femora were scanned with a Bruker SkyScan1172d (Billerica, MA) MicroCT scanner. Settings were 8.95 μm/voxel (70 kV, 142 μA, 420 ms, and 0.5 mm Al filter). Each bone was wrapped in saline soaked gauze, held in place with radiolucent foam, then placed in an enclosed container to prevent drying over the scan duration. Datasets were reconstructed using NRecon (Billerica, MA) software. In order to get bone density measurements, two phantoms of known density were scanned. The densities for the high and low density phantoms (diameter: 2 mm) were 0.25 and 0.75 g/cm3 calcium hydroxyapatite. Mean attenuation values for both phantoms were used in the CTAn software (Billerica, MA) built-in BMD calibration function to correlate attenuation with BMD.

**Fig. 2:**
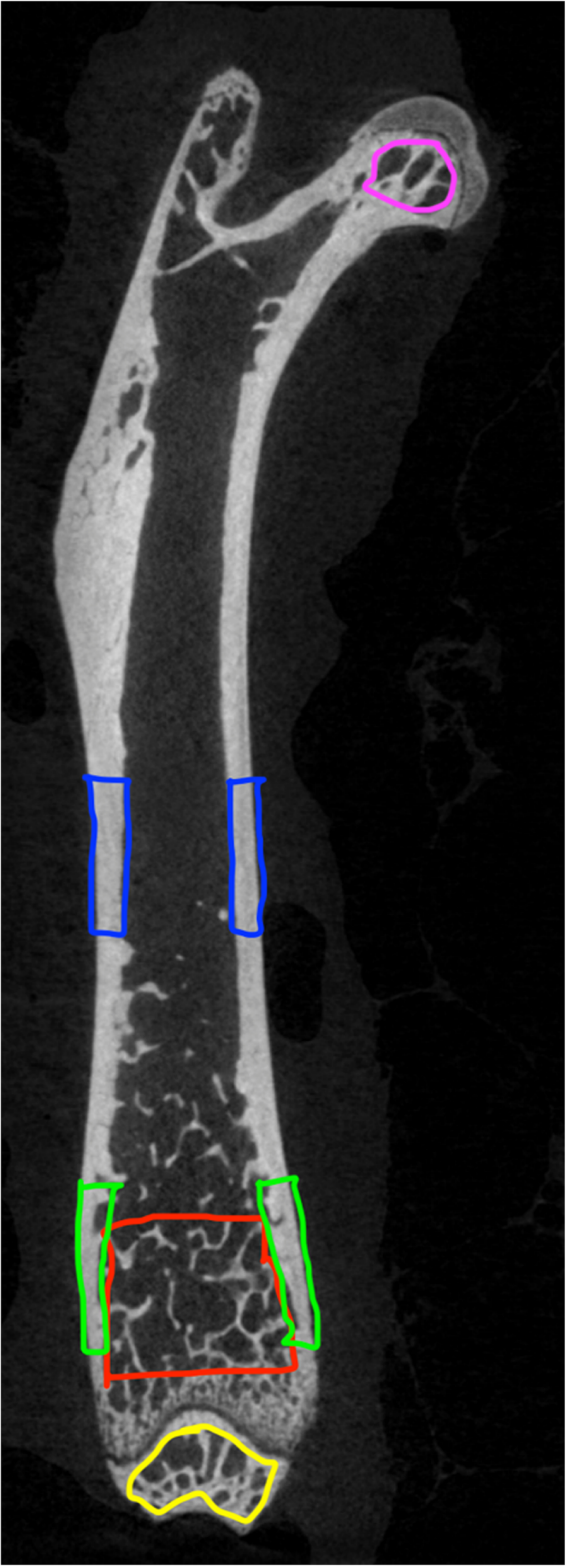
Cross-section of femur showing all 5 regions of interest: distal epiphyseal trabecular (yellow), distal metaphyseal trabecular (red), femoral head trabecular (purple), mid diaphysis cortical (blue), and distal metaphyseal cortical (green)

Five unique femoral regions of interest were defined with CTAn: distal epiphyseal trabecular, distal metaphyseal trabecular, femoral head trabecular, mid diaphysis cortical, and distal metaphyseal cortical as shown in Fig. 2.

Trabecular bone parameters were 3D: Tissue Volume (TV), Bone Volume (BV), Bone Volume Tissue Volume fraction (BV/TV), Bone Surface (BS), Bone Surface Bone Volume fraction (BS/BV), Trabecular Thickness (Tb.Th), Bone Volume fraction (BV/TV), Trabecular Separation (Tb.Sp), Trabecular Number (Tb.N), Structural Model Index (SMI), and Bone Mineral Density (BMD). Cortical bone parameters included 2D and 3D: Tissue Mineral Density (TMD), Bone Volume (BV), Tissue Volume (TV), Bone Volume/Tissue Volume (BV/TV), Bone Surface (BS), Bone Surface/Bone Volume fraction (BS/BV), Cortical Thickness (Ct.Th.), Moment of Inertia X (MOI X), Moment of Inertia Y (MOI Y), polar MOI, and Principle MOI max/min.

### Fluorochrome labels

Fluorochrome labels highlight areas of new bone apposition in vivo*.* Two different fluorochrome labels were administered at the beginning and end of weeks 0, 8, 16, and 24 in both LMMS and control groups. Calcein was administered at week 8 in all animals. Alizarin complexone was administered at: week 0 for the 8-week group, week 16 for the 16-week group, and week 24 for the 24-week group [23, 26]. These two labels of different colors were used in order to distinguish the labels upon analysis. Alizarin complexone and calcein were both subcutaneously administered in 1.4% isotonic sodium bicarbonate at concentration 10–20 mg/mL, at a dose of 15 mg/kg. Proper volume of dose was determined by weight of mouse on the day of each administration.

### Embedding/slicing/imaging

The same femur that was previously scanned with μCT was dehydrated in a graded ethanol series and embedded in poly methyl methacrylate (PMMA) resin dyed with 8 mg/mL sudan black. The resin was cured in a water bath at 37 °C for 1 week. The embedded bones were sectioned using a Jung RM2015 microtome (Leica, Germany). The microtome was mounted on a custom-built “Slicer” imaging system that included a microscope and UV source to view the embedded bone *en bloc.* The embedded bone was illuminated with a USHIO USH-102D mercury UV bulb (Tokyo, Japan). Images of the block were taken at 63x magnification, following each 5 μm thick slice.

A total of 520 5 μm-thick slices were taken starting at the onset of the distal epiphyseal trabecular bone, slicing distal to proximal. Between slices, an Optronics camera (Goleta, CA) in conjunction with Optronics Macrofire Picture Frame software was used to image the cross-sections of the specimen block with an exposure time of 9.76 s and a gain of 5.9688. Images of the growth plate were not taken.

### Image Deconvolution

Imaging a slice of bone in a mostly-opaque resin still introduces the challenge of removing background light. A solution was found by using deconvolution to analyze single labels and double labels only on the in-focus plane of bone. The “blind deconvolution function” from the Image Processing Toolbox in MATLAB R2016b (Natick, MA) was applied in order to deblur the microscopic images of the fluorescently labelled femoral cross-sections collected from the microtome slices of mouse cortical bone. This was done to remove any background florescence that could interfere with the in-plane fluorescence. The Gaussian function used a point spread function of size fspecial(‘gaussian’,10,7).

### Fluorochrome Histomorphometry

Three bones per group were sliced and analyzed. CalceinHisto software (Liverpool, UK) was used to perform histological analysis on 3 bone slices for all 3 regions of interest. The metaphyseal cortical region was defined as slice 518–520 i.e. the furthest distal slices taken, in order to analyze cortical bone. The metaphysical trabecular region started 172 slices proximal to the growth plate. The distal epiphyseal region started 48 slices distal to the start of the growth plate. These regions were selected for analysis because the μCT data suggested heightened remodeling activity at these sites. Images were uploaded to the software after undergoing deconvolution, then four steps were followed in order for CalceinHisto to calculate mineral apposition rate: outline, threshold, single and double label identification, and analysis. CalceinHisto calculated 3 important histological measurements: Mineral Apposition Rate (MAR), Mineralizing Surface to Bone Surface ratio (MS/BS), and Bone Formation Rate (BFR/BS). MAR (μm/day) was calculated as the distance between two labels divided by the time between label administration. MS/BS (%) was calculated as the ratio of label surface to bone surface. BFR/BS (μm^3^/μm^2^/day) was calculated as the product of MS/BS and MAR.

### Statistics

Time differences were found using a Tukey-Kramer test. LMMS vs SHAM differences were found at each time point. First unequal variance Welch’s test was done, if unequal variance, a two-tailed Student’s t-test was done, if equal variance, then ANOVA was done to determine statistical significance for two-sided and one-sided t tests. *P*-values less than 0.05 were highlighted with yellow in all tables, while *p*-values less than 0.1 were highlighted with orange in all tables. All statistical analysis were carried out with JMP software (SAS, Cary, NC).

## Results

All mice survived according to the study completion time posts, and were then scanned (MicroCT) and histology performed as described in the methods.

### MicroCT results

**Fig. 3:**
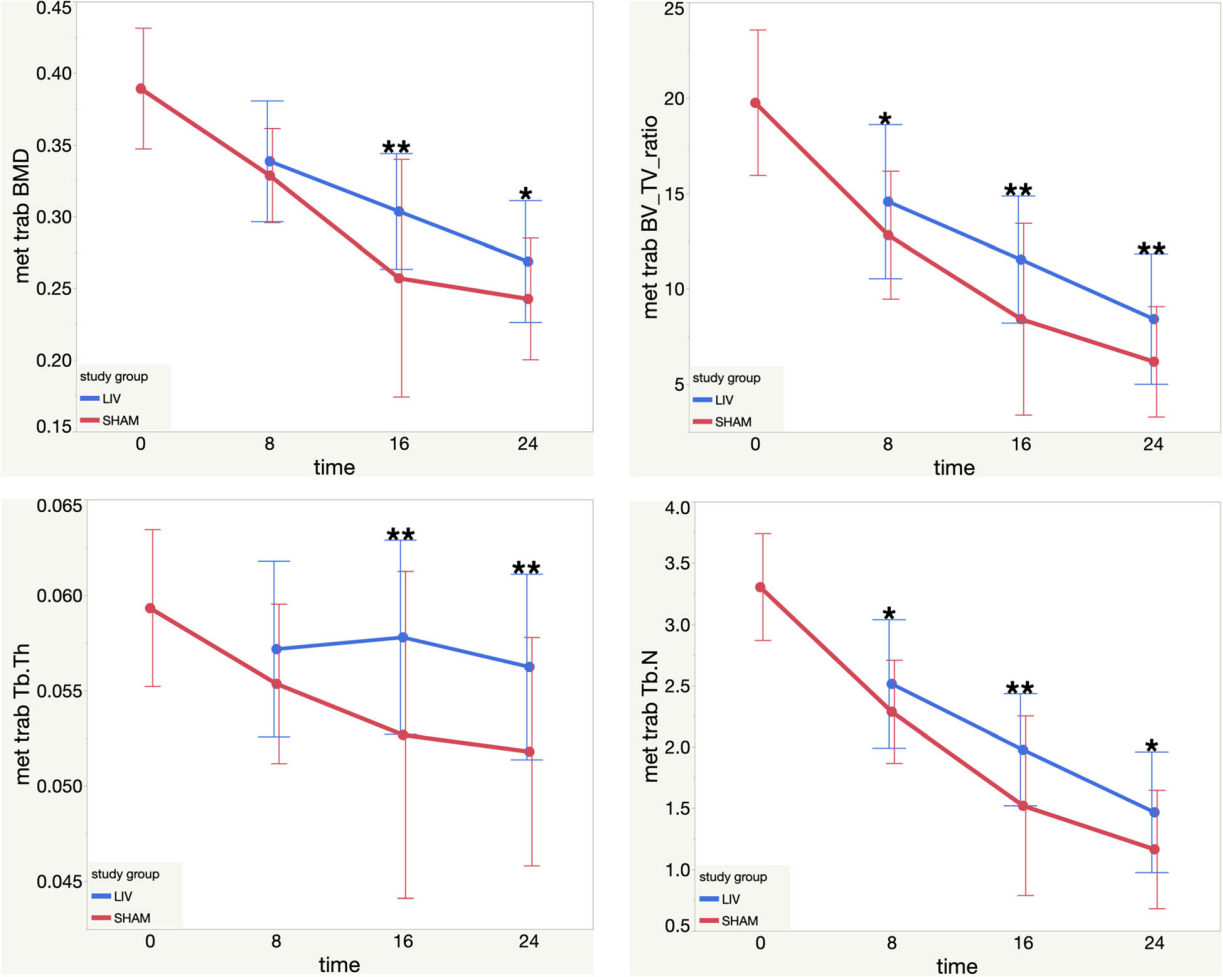
Metaphysis Trabecular MicroCT results. Top Left: Metaphysis Trabecular BMD mean values vs. time (post LMMS or Sham). Top Right: Metaphysis Trabecular BV-TV ratio mean values vs. time (post LMMS or Sham). Bottom Left: Metaphysis Trabecular Tb. Th mean values vs. time (post LMMS or Sham). Bottom Right: Metaphysis Trabecular Tb. N mean values vs. time (post LMMS or Sham). Each error bar is constructed using one standard deviation from the mean

**Fig. 4:**
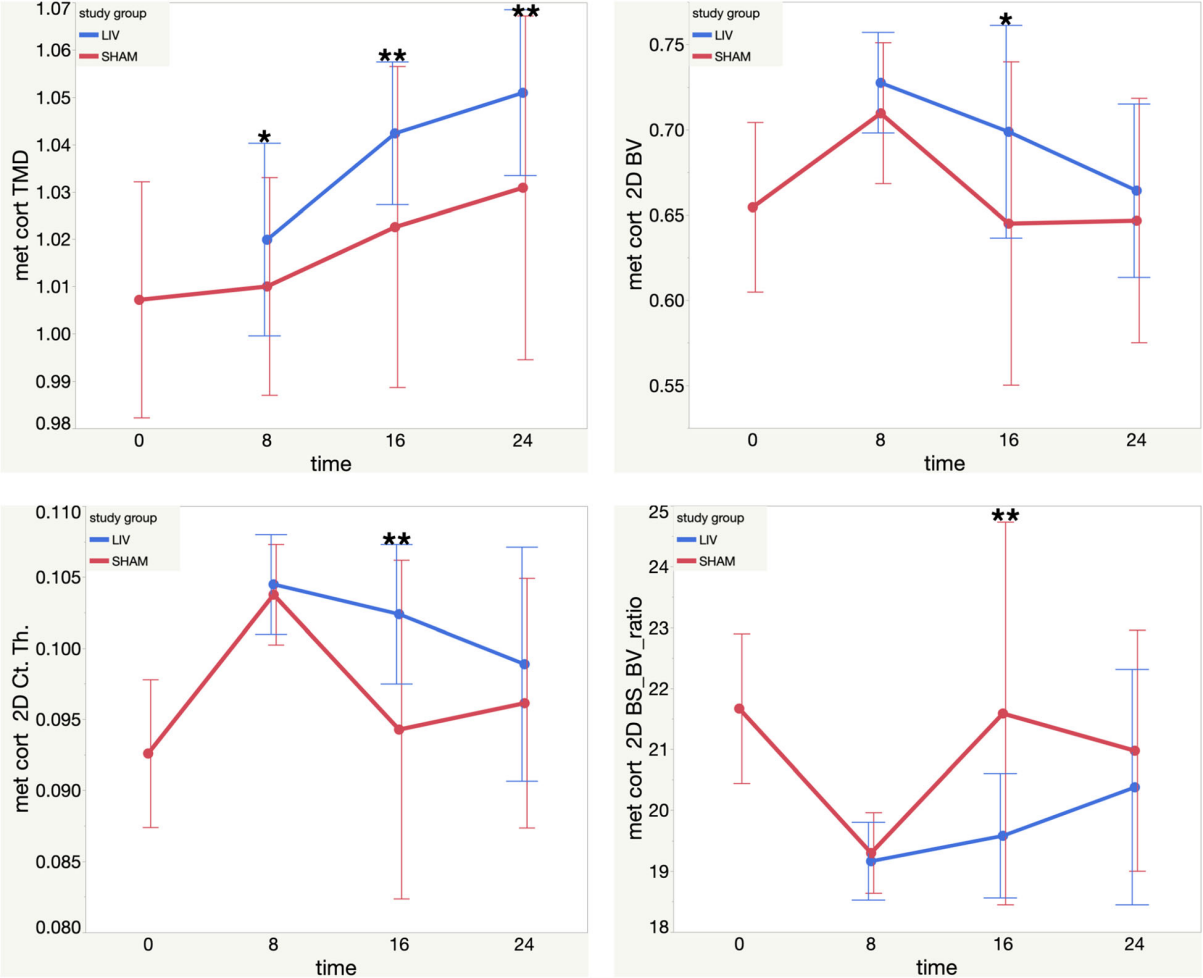
Metaphysis Cortical MicroCT results. Top Left: Metaphysis Cortical TMD mean values vs. time (post LMMS or Sham). Top Right: Metaphysis Cortical 2D BV mean values vs. time (post LMMS or Sham). Bottom Left: Metaphysis Cortical 2D Ct. Th mean values vs. time (post LMMS or Sham). Bottom Right: Metaphysis Cortical 2d BS-BV ratio mean values vs. time (post LMMS or Sham). Each error bar is constructed using one standard deviation from the mean. **(two-sided *p* < 0.05) *(two-sided *p* < 0.1)

Micro computed tomography (μCT) was used to quantify the morphological and BMD changes in bone across treatment groups. The cortical and trabecular ROI Micro-CT findings, including significance, are compiled in Table 1, Figs. 3, and 4.

**Table 1:**
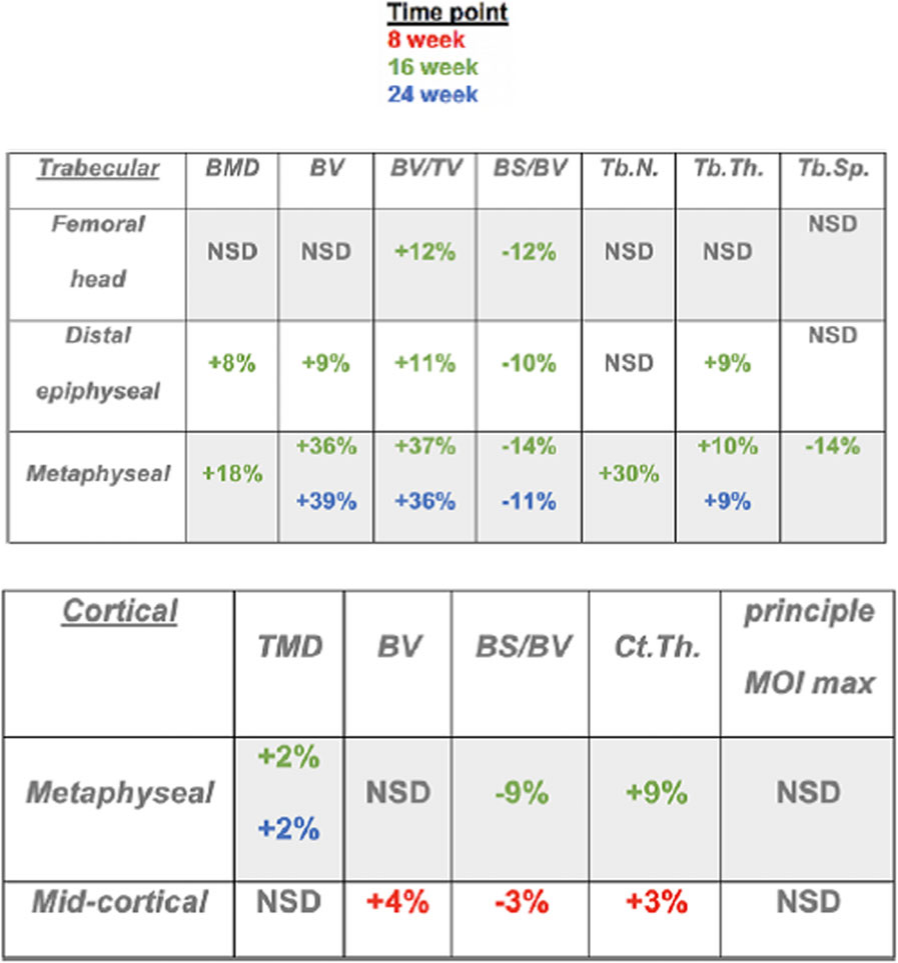
Cortical and trabecular μCT % differences. Positive sign indicates significant increases with LMMS compared to SHAM (*p* < 0.05). Colors correspond to time points. NSD means no significant differences. Bone mineral density (BMD), bone volume (BV), bone volume/tissue volume (BV/TV), bone surface/tissue volume (BS/TV), trabecular number (Tb.N.) trabecular thickness (Tb.Th.), trabecular spacing (Tb.Sp.), tissue mineral density (TMD), cortical thickness (Ct.Th.), principle moment of inertia (MOI) maximum.

#### Distal epiphysis trabecular ROI

In the distal epiphyseal trabecular region, there were no significant differences between LMMS and SHAM at 8 weeks. At 16 weeks, LMMS increased trabecular BMD and parameters that indicate robust trabecular bone compared to SHAM. These parameters include: BMD (7.7% or 1.2 effect size), BV/TV (11.4% or 1.3 effect size), and Tb.Th. (8.9% or 1.2 effect size) *p* < 0.05. Furthermore, higher BS/BV (10.1% or 2 effect size) in the SHAM group compared to the LMMS group could mean thinner trabeculae. By 24 weeks, there were trends in the above parameters that indicated the effects of LMMS at 16 weeks remained but were slightly diminished.

#### Femoral head results

There were no treatment differences at 8 weeks in the femoral head trabecular bone region. At 16 weeks the LMMS group trended higher BMD, Tb.Th., and Tb. N, compared to the SHAM group. There was a significant increase in BV/TV (12.4% or 1.3 effect size) at 16 weeks in the LMMS group compared to SHAM *p* < 0.05. These findings along with significantly higher BS/BV (11.7% or 1.9 effect size) in the SHAM group, all suggest robust trabecular bone in the LMMS group compared to the SHAM group *p* < 0.05. There were only trends at 24 weeks, with higher BMD, BV/TV, Tb.Th., and Tb.N. for the LMMS group compared to the SHAM group.

#### Metaphysis trabecular

At 16 weeks, BMD in the LMMS group was 18.2% or 1.15 effect size significantly greater compared to the SHAM group (*p* < 0.05). At 24 weeks, there was a trend of 10.8% or 0.6 effect size greater BMD in the LMMS group compared to the SHAM group (two-sided *p* < 0.1 one-sided *p* < 0.05). Results are shown in Fig. 3.

At 8 weeks, there was a 4.6 or 0.4 effect size trend increase in the SHAM group compared to the LMMS group (one-sided *p* < 0.1). At 16 weeks, BS/BV was 14.3% or 1.6 effect size significantly larger in the SHAM group compared to the LMMS group (*p* < 0.05). The effect continued through 24 weeks with a 10.6% or 1 effect size (*p* < 0.05).

At 16 and 24 weeks, Bone surface (BS) trended 24.79% 0.88 effect size and 26.51% or 0.68 effect size respectively in the LMMS group compared to the SHAM group (two-sided *p* < 0.1 one-sided *p* < 0.05).

LMMS had significantly greater bone volume (BV) compared to SHAM at 16 weeks by 36.08% 0.87 effect size and at 24 weeks by 39.35% 0.68 effect size (*p* < 0.05). There were no treatment effects for tissue volume (TV) in the metaphyseal trabecular region.

At 8 weeks, the LMMS group had a trend of 13.7% or 0.4 effect size higher BV/TV compared to the SHAM group (one-sided *p* < 0.1). At 16 weeks, the LMMS group had a 37.1% or 0.9 effect size greater BV/TV compared to the SHAM group (*p* < 0.05). Additionally at 24 weeks, the LMMS group had a 36.4% or 0.65 effect size increase in BV/TV compared to the SHAM group (*p* < 0.05). Results are shown in Fig. 3.

At 8 weeks, LMMS trended 11.1% or 0.6 effect size greater intersecting surface (i.S.) compared to SHAM (one-sided *p* < 0.1). This trend continued at 16 weeks with a 18.4% or 0.7 effect size (one-sided *p* < 0.1). In addition at 24 weeks, LMMS significantly increased i.S. 25.5% or 0.7 effect size compared to SHAM (*p* < 0.05).

SHAM trended greater structural model index (SMI) compared to LMMS at: 8 weeks (5.8%, 0.5 effect), 16 weeks (9.6%, 0.8 effect), and 24 weeks (6.2%, 0.5 effect) (one-sided *p* < 0.05).

At 8 weeks, the LMMS group trended 10% or 0.4 effect size greater trabecular number (Tb.N) compared to the SHAM group (one-sided *p* < 0.1). At 16 weeks, the LMMS group had 30% or 1 effect size significantly greater (Tb.N) compared to the SHAM group (*p* < 0.05). At 24 weeks, LMMS trended 26% or 0.6 effect size greater compared to SHAM (two-sided *p* < 0.1, one-sided *p* < 0.05). Results are shown in Fig. 3.

At 8 weeks, the SHAM group had a trend of 10.8% or 0.4 effect size greater trabecular pattern factor (Tb.Pf) compared to the LMMS group (one-sided *p* < 0.1). At 16 weeks, the SHAM group had a significant 24.5% or 1.3 effect size greater compared to the LMMS group (*p* < 0.05). This difference remained at 24 weeks, but was reduced to 16.3% or 0.8 effect size (*p* < 0.05).

At 16 weeks, the SHAM group had a 14.4% or 1.4 effect size significantly greater trabecular separation (Tb.Sp.) compared to the LMMS group (*p* < 0.05). At 24 weeks, the SHAM group trended 10.1% or 0.9 effect size greater than the LMMS group (one-sided *p* < 0.1).

Trabecular thickness (Tb.Th.) significantly increased in the LMMS group compared to the SHAM group at both 16 and 24 weeks by 9.7% or 1 effect size and 8.6% or 0.9 effect size respectively (*p* < 0.05). Results are shown in Fig. 3.

Contrary to the other trabecular regions, there was evidence of LMMS effects at 8 weeks in the metaphyseal trabecular region. These include trends of increased BV/TV, Tb.N., and i. S in the LMMS group. Furthermore, higher BS/BV, SMI, and Tb.Pf. 8-week trends were found in the SHAM group compared to the LMMS group. At 16 weeks there were significant increases in BMD, BV/TV, Tb.Th., Tb.N., and trends of increased i. S in the LMMS group compared to SHAM. Similar to 8 weeks, BS/BV, Tb.Pf., and Tb.Sp. were significantly higher in the SHAM group compared to LIV. Differences were still significant at 24 weeks for BS/BV, BV/TV, Tb.Th., Tb.Pf., and i.S. Trends were seen for BMD, SMI, and Tb.N.

#### Metaphyseal cortical bone

At 8 weeks, LMMS TMD trended 0.98% or 0.49 effect size greater compared to SHAM (one-sided *p* < 0.1). At 16 weeks, LMMS TMD was significantly greater by 1.94% or 1.31 effect size compared to SHAM (*p* < 0.05). This difference remained at 24 weeks, with a 1.95% or 1.15 effect size greater TMD (*p* < 0.05). Results are shown in Fig. 4.

At 16 weeks, cortical thickness (Ct.Th.) was 8.62% or 1.65 effect size significantly greater in the LMMS group compared to the SHAM group (two-sided *p* < 0.1). Results are shown in Fig. 4.

At 16 weeks, BS/BV was 9.3% or 1.96 effect size significantly greater in the SHAM group compared to the LMMS group (*p* < 0.05). Results are shown in Fig. 4.

At 16 weeks, the LMMS group trended 9.95% or 0.7 effect size greater 2D mean MOI Y compared to the SHAM group (two-sided *p* < 0.1, one-sided *p* < 0.05).

2D principle MOI trended 5.64% or 0.64 effect size greater in the LMMS group compared to the SHAM group at 8 weeks (one-sided *p* < 0.1). This trend was seen at 16 weeks as well, with a 7.36% or 0.48 effect size (one-sided *p* < 0.1). At 16 weeks, 2D mean principle MOI trended 10.4% or 0.72 effect size larger in the LMMS group compared to the SHAM group (two sided *p* < 0.1, one-sided *p* < 0.05).

At 16 weeks, 2D cortical bone volume (BV) in the LMMS group trended 8.35% or 0.86 effect size greater compared to the SHAM group (two sided *p* < 0.1, one-sided *p* < 0.05). Results are shown in Fig. 4.

At 16 weeks, 2D porosity was 35.25% or 1.389 effect size greater in the SHAM group compared to the LMMS group (*p* < 0.05).

At 8 weeks there was a trend of higher TMD in the LMMS group compared to the SHAM group. At 16 weeks, the LMMS group metaphyseal cortical region had significantly higher TMD, Ct.Th., and porosity compared to the SHAM group. At 16 weeks, the SHAM group had significantly higher BS/BV compared to the LMMS group. There were trends of higher BV and moment of inertias including: mean MOI Y, mean principle MOI max, mean principle MOI min, and MOI Y in the LMMS group compared to SHAM. At 24 weeks, TMD in the LMMS group was significantly higher compared to the SHAM group. No other trends were observed in the 24-week group.

#### Middle cortical diaphysis

At 8 weeks, the SHAM group had 3.07% or 0.93 effect size significantly greater BS/BV compared to the LMMS group (*p* < 0.05). At 8 weeks, the LMMS group had 4.26% or 0.68 effect size greater BV compared to SHAM (*p* < 0.05). At 8 weeks, cortical thickness (Ct.Th.) was 3.18% or 0.89 effect size larger in the LMMS group compared to the SHAM group (*p* < 0.05).

Differences in the middle cortical region only occurred at 8 weeks. There were no TMD differences. The LMMS group had significantly higher Ct.Th. and BV while BS/BV was higher in the SHAM group.

### Dynamic Histomorphology results

**Fig. 5:**
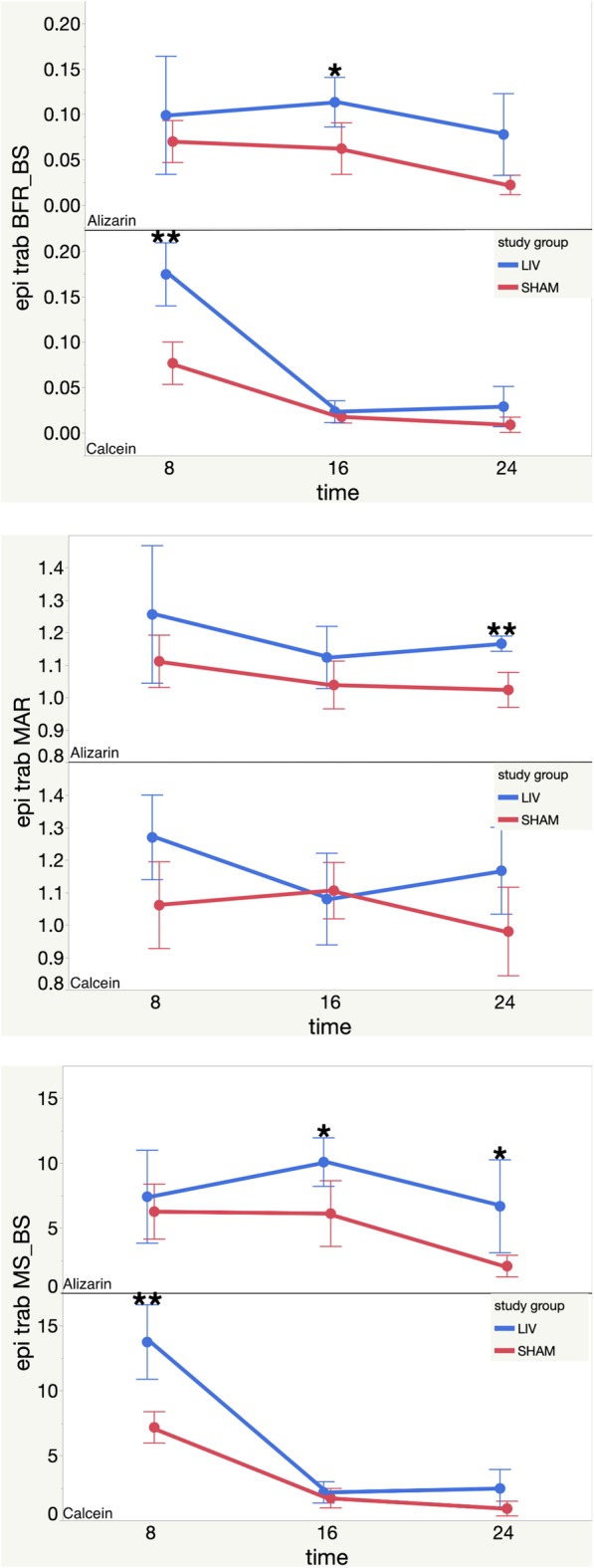
Epiphyseal trabecular histomorphology results. Top: Metaphysis Trabecular BFR-BS mean values for Alizarin and Calcein vs. time (post LMMS or Sham). Middle: Metaphysis Trabecular MAR mean values for Alizarin and Calcein vs. time (post LMMS or Sham). Bottom: Metaphysis Trabecular MS-BS mean values for Alizarin and Calcein vs. time (post LMMS or Sham). Each error bar is constructed using one standard deviation from the mean. **(two-sided *p* < 0.05) *(two-sided *p* < 0.1)

**Fig. 6:**
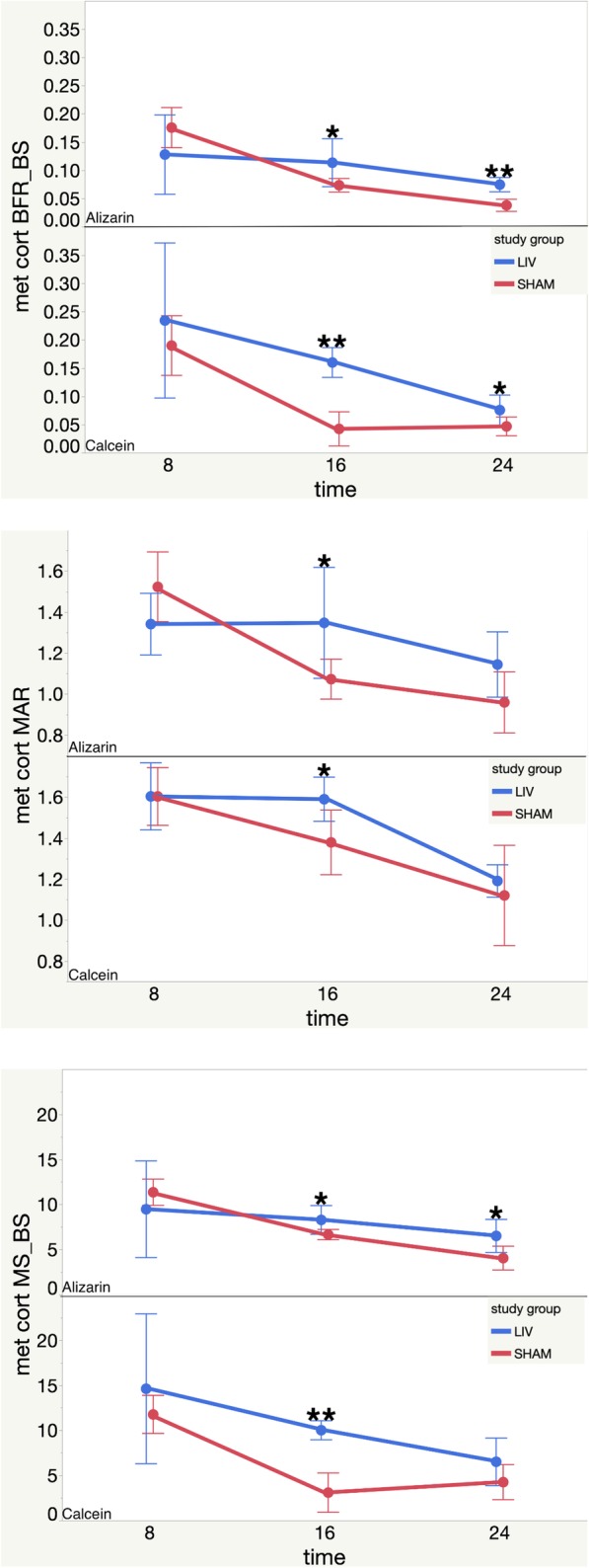
Metaphyseal cortical histomorphology results. Top: Metaphysis Cortical BFR-BS mean values for Alizarin and Calcein vs. time (post LMMS or Sham). Middle: Metaphysis Cortical MAR mean values for Alizarin and Calcein vs. time (post LMMS or Sham). Bottom: Metaphysis Cortical MS-BS mean values for Alizarin and Calcein vs. time (post LMMS or Sham). Each error bar is constructed using one standard deviation from the mean. **(two-sided *p* < 0.05) *(two-sided *p* < 0.1)

Dynamic histomorphology was completed to quantify bone apposition throughout the study and compare how time and treatment affected bone histomorphometry. Both calcein and alizarin single and double labels were visible in femoral cross sections, which indicated all 4 label administrations were successful. Three bones per group were studied. Dynamic histomorphology results are summarized in Table 2, Figs. 5, and 6. This includes all significant LMMS vs SHAM percent difference effects at each time point for all 3 ROIs.

**Table 2:**
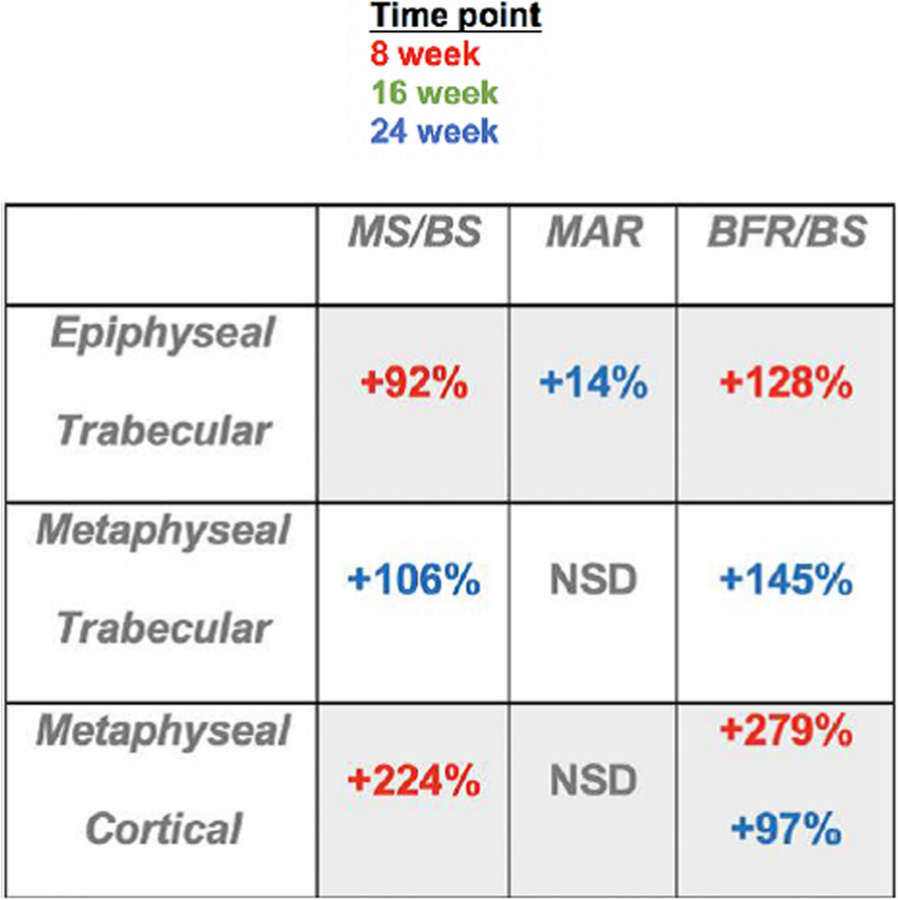
Dynamic histomorphology % differences between LMMS and SHAM. Positive sign indicates significant increase with LMMS compared to SHAM (*p* < 0.05). Colors correspond to time points. NSD means no significant differences. Mineral surface/bone surface (MS/BS), mineral apposition rate (MAR), bone formation rate/bone surface (BFR/BS).

#### Epiphyseal trabecular

At 8 weeks, BFR/BS was 127.54% or 2.82 effect size significantly greater in the LMMS group compared to the SHAM group (*p* < 0.05). At 16 weeks, BFR/BS trended 82.14% or 1.87 greater in the LMMS group compared to the SHAM group (two-sided *p* < 0.1 one-sided *p* < 0.05). At 24 weeks, BFR/BS trended 250.00% or 1.23 effect size greater in the LMMS group compared to the SHAM group (one-sided *p* < 0.1). Results are shown in Fig. 5.

At 8 weeks, MAR trended 19.70% or 1.6 effect size greater in the LMMS group compared to SHAM (one-sided *p* < 0.1). At 24 weeks, MAR was significantly greater in the LMMS group by 13.91% or 6.05 effect size compared to the SHAM group (*p* < 0.05). Results are shown in Fig. 5.

At 8 weeks, MS/BS was 91.70% or 2.29 effect size significantly greater in the LMMS group compared to the SHAM group (*p* < 0.05). At 16 weeks, MS/BS trended 64.90% or 2.12 effect size larger in the LMMS group compared to the SHAM group (two-sided *p* < 0.1 one-sided *p* < 0.05). This trend continued at 24 weeks as 223.64% or 1.29 effect size (two-sided *p* < 0.1 one-sided *p* < 0.05). Results are shown in Fig. 5.

Epiphyseal trabecular MS/BS was significantly greater in the LMMS group compared to the SHAM group at 8 weeks by 91.70% or 2.29 effect size. A trend of greater MS/BS was observed in the LMMS group at both 16 and 24 weeks. MAR was significantly greater in the LMMS group at 24 weeks only by 13.9% or 6.05 effect size, although there was a trend at 8 weeks. BFR/BS was significantly greater in the LMMS group at 8 weeks by 127.54% or 2.82 effect size, and there was a trend at both 16 and 24 weeks.

#### Metaphyseal cortical

For the 8-week label in the 16-week group, LMMS had 278.95% or 4.45 effect size greater BFR/BS compared to SHAM (*p* < 0.05). No significant differences between LMMS and SHAM were found at 16 weeks. However at 24 weeks, the LMMS group had a 97.06% or 2.91 effect size greater BFR/BS compared to the SHAM group (*p* < 0.05). Results are shown in Fig. 6.

No LMMS vs SHAM significant differences in MAR were found at any time point. There were trends of 15.27% or effect size 1.95 of increased MAR with LMMS for the 8-week label in the 16-week group (one-sided *p* < 0.1). The trend remained at 16 weeks (25.6% or 1.01 effect size) (one-sided *p* < 0.1). Results are shown in Fig. 6.

For the 8-week label in the 16-week group, the LMMS group had 223.73% or 6.56 effect size significantly greater MS/BS compared to the SHAM group (*p* < 0.05). The LMMS group trended greater at both 16 and 24 weeks, 24.42% or 1.02 effect size, and 60.75% or 1.34 effect size, respectively, compared to the SHAM group (one-sided *p* < 0.1). Results are shown in Fig. 6.

MS/BS was significantly higher at 8 weeks for the 16-week group with LMMS compared to SHAM by 223.73% or 6.56 effect size. Only a trend of higher LMMS compared to SHAM continued at 16 and 24 weeks. MAR only trended higher with LMMS at 8 weeks for the 16-week group, and at 16 weeks. BFR/BS was significantly higher by 278.95% or 4.45 effect size at 8 weeks for the 16-week group, trended higher at 16 weeks, then was significantly higher at 24 weeks in the LMMS group by 97.06% or 2.91 effect size compared to the SHAM group.

#### Metaphyseal trabecular

There were no 8 or 16-week BFR/BS differences between LMMS and SHAM groups. At 24 weeks, the LMMS group had 145.16% or 2.98 effect size significantly greater BFR/BS compared to the SHAM group (*p* < 0.05).

No LMMS vs. SHAM significant differences in MAR were found at any time point. One trend of 16.19% or 1.13 effect size was found in the LMMS group compared to the SHAM group (one-sided *p* < 0.1).

At 8 weeks, there was a 15.43% or 1.16 effect size trend of greater MS/BS in the LMMS group compared to the SHAM group (one-sided *p* < 0.1). No differences were found at 16 weeks. At 24 weeks, LMMS had 105.71% or 3.93 effect size significantly higher MS/BS compared to the SHAM group (two-sided *p* < 0.05). In the metaphyseal trabecular region, MS/BS trended higher at 8 weeks in the LMMS group compared to the SHAM group, then at 24 weeks, MS/BS was significantly greater in the LMMS group by 105.71% or 3.93 effect size compared to the SHAM group. Only one trend was observed for MAR which was at 24 weeks. In addition, BFR/BS was significantly greater in the LMMS group by 145.16% or 2.98 effect size compared to the SHAM group at 24 weeks only.

## Discussion

### Trabecular region μCT

Micro computed tomography (μCT) was used to quantify the morphological and BMD changes in bone across treatment groups. In the trabecular regions, after 8 weeks of LMMS, it was expected that the LMMS group would have significantly more bone and more robust trabeculae compared to the SHAM group. However, this was generally not the case for the three regions of interest studied although there was an 8-week trend of higher bone volume, bone volume ratio, and trabecular number for LMMS compared to SHAM in the femoral metaphyseal trabecular region only. Previous studies have found that some effects of LMMS were apparent immediately after treatment [22, 23, 25, 27]. These included increased bone volume and tissue volume by 10.5 and 13.7% respectively in the murine tibial trabecular metaphysis after 6 weeks of LMMS, but no difference in BV/TV [23]. No difference in BV/TV for trabecular bone could be interpreted as no gain in trabecular bone. Shorter studies that applied 3–5 weeks of LMMS, did not yield differences in bone volume or trabecular parameters quantified with μCT [22, 25]. In the current study we did not find significant increases in trabecular bone immediately after LMMS, similarly to previous studies. The dynamic histomorphology results could potentially explain the lack of immediate effects of LMMS. Due to high amounts of remodeling at 8 weeks, the new bone could have been in the process of mineralization which may not be visible in μCT.

8 weeks after LMMS cessation, significant increases in bone volume, BMD, and other trabecular parameters compared to SHAM were apparent in all three ROIs studied. The 16-week LMMS group had significantly higher BMD, BV, BV/TV, Tb.Th., and Tb.N. compared to the SHAM group. In addition, the SHAM group had significantly higher BS/BV compared to LMMS, which further indicates thicker bone after LMMS treatment. These parameters increased over a range of 7.7–37.1% or 0.9–1.6 effect size. This clearly illustrates both increased bone amount and quality of structure attributed to LMMS.

As far as we are aware, there are no animal studies that looked at the longer-term residual effects of LMMS. The delayed LMMS benefit shown by μCT in this study could explain why previous studies did not find sufficient significant differences; the bone could be in the process of mineralization or other phenomenological processes. No animals were studied between 8 and 16 weeks, so the rate and behavior of increased modeling immediately post LMMS is unknown.

Effects of LMMS were still measurable 16 weeks post LMMS. Trends in the distal epiphyseal and femoral head region were observed and significant effects remained in the metaphyseal trabecular region at 24 weeks. These included: BV/TV, BV, Tb.Th., and BS/BV. Effects were similar compared to the 16-week group with a range of 8.6–39.4% or 0.7–1.0 effect size differences. Finding differences at 24 weeks – which was double the time that the mice received LMMS -- indicates that there are long term residual effects of LMMS. The study ended at 24 weeks, so it is unknown how long these positive effects last or if SHAM and LMMS bones become indistinguishable after some time.

### Cortical region μCT

Using μCT in the cortical regions, after 8 weeks of LMMS it was expected that the LMMS group would have significantly more and thicker bone compared to the SHAM group. This was the case in the mid diaphyseal cortical region only. There were increased BV and Ct.Th., and decreased BS/BV in the LMMS group compared to the SHAM group. The differences ranged from 3.1–4.5% or 0.7–0.9 effect size. No significant differences or trends were found at 16 or 24 weeks. The mid diaphyseal region was the only cortical or trabecular region that had significant LMMS effects at 8 weeks. These data suggest mid cortical bone may have a shorter response time to the effects of LMMS compared to trabecular regions.

No significant differences were seen at 8 weeks in the metaphyseal cortical region, however, the 8-week LMMS group trended higher TMD and MOI max compared to the SHAM group. Xie et al. found a 29% increase in tibial metaphyseal MOI max after 6 weeks of LMMS, whereas the current study only found a trend of 6% [23]. Some potential reasons for the discrepancies could include bone studied, LMMS regimen, and age differences. Significant differences between LMMS and SHAM were measured at 16 weeks including TMD, Ct.Th., and BS/BV. The differences ranged from 1.9–9.3% or 1.3–2.0 effect size. Not unlike the metaphyseal trabecular region, the metaphyseal cortical region had significant increases in bone parameters including density 8 weeks post LMMS. This metaphyseal region seems to have the most bone formation due to LMMS compared to the other regions studied. It may be due to proximity to the growth plate, which was active in the young mice used in the study. At 24 weeks, TMD was significantly higher with LMMS treatment, nearly the same magnitude difference found in the 16-week group. Unlike the 16-week group, there we no geometric differences between LMMS and SHAM at 24 weeks.

One limitation of this study was not having a group beyond 24 weeks. In some instances, LMMS effects were still seen at 24 weeks, so in order to find a point where groups were no longer different, mice need to stay on study longer. In order to facilitate this, in vivo μCT could be used to track individual bone parameters over time. In vivo μCT would also allow for individual tracking of bone formation and resorption over time plus reduction of animals used during the study. Only females were studied, so sex differences are unknown. Femurs were studied because they are better suited for mechanical testing. Tibias have been used in other mouse LMMS studies, so comparing results in this study to those proves somewhat difficult [22, 23, 25, 28].

A limitation of this study overall is that this is a mouse model, and mouse and human bones are different. One difference is that mice do not undergo secondary osteonal remodeling [29]. This means that mice only form new bone, or resorb old bone, not replace existing bone. Another difference is human cortical bone is organized by forming primary osteons, which have circumferential lamellar bone around a Haversian canal at the center [30]. Murine cortical bone doesn’t form osteons, but rather organized lacunae and canaliculi that align with collagen matrix fiber direction [31]. In mouse cortical bone femoral cross sections, newer bone on the periosteal and endosteal surfaces are more organized compared to the middle layer visible under polarized microscopy [31]. Due to these differences, the results found in this study may not translate fully to human bone.

Both cortical and trabecular bone μCT analysis resulted in significant morphometric parameters and bone density changes with LMMS treatment. These differences were mostly 8 weeks after LMMS cessation, but were also detected 16 weeks post cessation, and immediately after the LMMS regimen. The case has been made that LMMS could be used as a clinical therapy to increase bone formation for people at risk for osteoporosis or for children with ambulation disabilities [1, 9]. It is well known that weight bearing activities during adolescence increases bone mass and these effects can be detected even 8 years after these activities [32, 33]. Strong bone can be built during the window of opportunity in development before growth plates fuse and longitudinal growth stops [32, 34]. Since skeletal maturity is usually reached by the end of the second decade, these interventions may be more effective if they take place before, to get the most benefit from LMMS. Such was found in adolescent females who underwent LMMS had greater improvements in BMD compared to a separate study of the effects of LMMS on postmenopausal women [13, 14].

### Dynamic Histomorphology

Dynamic histomorphology was completed to quantify bone apposition throughout the study and compare how time and treatment affected MS/BS, MAR, and BFR/BS. Both calcein and alizarin single and double labels were visible in femoral cross sections, which indicated all 4 label administrations were successful. Three bones per group were studied. It was hypothesized that LMMS would increase bone apposition rate compared to age matched controls during and after LMMS therapy.

At 8 weeks, there was a significant effect of LMMS observed as increased MS/BS and BFR/BS in the epiphyseal trabecular region and metaphyseal cortical region. This aligns with the findings of Xie et al., which showed significantly increased BFR/BS at 3 weeks and MS/BS at 6 weeks of LMMS in the mouse tibia [22, 23]. Although the LMMS regimen was different from the current study, all found LMMS benefits histologically, showing mouse bone responding to LMMS from 3 to 8 weeks. The lack of μCT morphological findings in Xie et al. 3-week study, and some cortical benefits in Xie et al. 6-week study shows a delayed effect between histological and μCT morphological findings [22, 23]. Furthermore, the current study supports this idea of a delayed response between histology and μCT findings.

There were no significant differences observed for any of the histological parameters at all regions of interest in the 16-week group, 8 weeks following the end of the LMMS and SHAM treatments. The LMMS group trended higher in the metaphyseal cortical region compared to the SHAM group for all three parameters. The lack of significant LMMS effects on histology 8 weeks after LMMS stopped is reasonable; there was not more active remodeling occurring since the LMMS stopped.

Contradicting evidence was found 16 weeks post LMMS cessation (24-week group). Significant LMMS effects on MS/BS, MAR, and BFR/BS were found, especially in the metaphyseal trabecular region, where both MS/BS and BFR/BS were significantly greater in the LMMS group compared to the SHAM group. Additionally, the only instance where LMMS significantly affected MAR was in the 24-week epiphyseal trabecular region. It is compelling that LMMS has an effect on active bone formation long term, and all significant and trend effects in this study benefit LMMS compared to SHAM. The effect of LMMS may be diminished 16 weeks post LMMS cessation because the effects of LMMS may wear off, since bone adapts to the loads it experiences [35]. The diminished effect could also be attributed to age, as bone may not be as reactive to mechanical stimuli as the mice age. The amount of time (16 weeks) could be nearing the end of the long-term effects of LMMS in mice during this study. Many factors could contribute to this time period including: LMMS parameters, age during treatment, age after treatment, sex, and activity level. It appears that similar to other musculoskeletal tissues, mechanical stimuli can have long-term effects, but they eventually diminish. For example, physical therapy can be an effective treatment for musculoskeletal injuries, but only if the patient keeps doing the exercises and stretches. Once the patient stops, the effects will start to fade with time.

One limitation was that the alizarin labels did not fluoresce as brightly as the calcein labels. This sometimes made it difficult to find alizarin labels. The darkness of the resin was good at preventing out of plane fluoresce, but it made the image very dark overall, which made it difficult to determine the outline of the bone and segmentation from the resin.

## Conclusion

This study showed that LMMS treatment has long term effects on bone structure. Mice who received an 8-week treatment of LMMS immediately had improved cortical bone morphological parameters but not TMD in the middle cortical region only. In regard to the first hypothesis -- to see if LMMS treatment will improve trabecular and cortical bone morphological parameters including BMD compared to age matched controls during and after LMMS therapy -- at time points 8 and 16 weeks post LMMS, both cortical and trabecular regions did have improved morphological parameters and bone density compared to age matched controls. To address the second hypothesis -- to see if LMMS treatment will increase bone apposition rate compared to age matched controls during and after LMMS therapy -- LMMS did increase bone apposition rate compared to aged matched controls immediately after and 16 weeks post LMMS, but not 8 weeks post LMMS. These results improve our understanding of the long-term residual effects of LMMS and will be helpful in developing treatment strategies to increase bone health in younger individuals.

Our culture historically tends to deal with problems as they arise, instead of preventing them before they occur. This work has greater implications in preventative medicine. Preventative medicine is the future, and with rising costs in healthcare, it only makes sense to do our best to prevent disease. This only works if there are early predictors of disease, and with better understanding of etiology and genetics, this could be possible.

## Data Availability

The datasets used and/or analyzed during the current study are available from the corresponding author on reasonable request.
